# Prolactin level changes according to atypical antipsychotics use: a study based on Clinical Data Warehouse

**DOI:** 10.1192/j.eurpsy.2024.506

**Published:** 2024-08-27

**Authors:** I. Sohn, J.-H. Jeong

**Affiliations:** ^1^Psychiatry, Keyo Hospital, Uiwang; ^2^Psychiatry, St. Vincent’s Hospital, College of Medicine, The Catholic University of Korea, Suwon, Korea, Republic Of

## Abstract

**Introduction:**

Antipsychotics are associated with increased serum prolactin. It depends on the type of the antipsychotics and gender. There are previous studies, but it is necessary to compare them including new drugs.

**Objectives:**

Antipsychotic drugs are known as the major cause of non-neoplastic hyperprolactinemia. This study aimed to investigate the levels of serum prolactin elevation depending on the use of antipsychotic drugs in patients through the Clinical Data Warehouse

**Methods:**

Our study included 118 subjects who were all diagnosed according to ICD-10 for schizophrenia, schizotypal and delusional disorders, manic episodes, and bipolar affective disorders. All the subjects were taking one of risperidone, blonanserin, amisulpride, and olanzapine. They had prolactin blood tests collected retrospectively through CDW.

**Results:**

Among the 118 subjects included in the analysis, the mean serum prolactin level was 65.1± 54.7ng/ml. Serum prolactin levels were significantly higher in subjects taking risperidone or amisulpride compared to blonanserin and olanzapine. The female subjects who took amisulpride or olanzapine had significantly higher prolactin levels, but there was no difference in prolactin levels between the sex in the subjects who took risperidone or blonanserin.

**Conclusions:**

This study suggests the need for regular monitoring of serum prolactin levels in patients who are taking antipsychotics, especially in female patients. Further studies on the subjects with controlled confounding variables and larger sample groups are needed.

**Disclosure of Interest:**

None Declared

